# Amelioration of insulin resistance in rat cells by Astragalus polysaccharides and associated mechanisms

**DOI:** 10.3892/etm.2014.1626

**Published:** 2014-03-18

**Authors:** HONGZHI LIU, JIANMEI BAI, XIAOGANG WENG, TAO WANG, MEIJUAN LI

**Affiliations:** 1 Department of Internal Medicine, Tianjin Prevention and Treatment Center of Occupational Diseases, Tianjin 300011, P.R. China; 2 Department of Internal Medicine, Xinxing Hospital of Tianjin, Tianjin 300070, P.R. China; 3 Department of Endocrinology, The First Affiliated Hospital of Xinxiang Medical University, Weihui, Henan 453100, P.R. China

**Keywords:** Astragalus polysaccharides, ameliorating, insulin resistance, mechanism

## Abstract

The aim of this study was to investigate the function of Astragalus polysaccharides (APS) in ameliorating insulin resistance (IR) in rat cells and to elucidate the associated mechanisms. Fully differentiated, induced 3T3-L1 rat adipocytes were divided into a control group and three intervention groups. The intervention groups were incubated in media containing 0.001, 0.1 and 10 μg/μl APS, respectively, for 48 h. Following treatment, levels of interleukin (IL)-6 and adiponectin secreted by the cultured adipocytes were measured using enzyme-linked immunosorbent assay. Levels of adiponectin secreted by the 3T3-L1 adipocytes in the moderate-concentration intervention group were significantly increased compared with those in the control group (P<0.05), whereas levels of adiponectin secreted by the 3T3-L1 adipocytes in the low- and high-concentration intervention groups were decreased compared with those in the control group (P<0.05 and P>0.05, respectively). Levels of IL-6 secreted by the 3T3-L1 adipocytes in the three intervention groups were lower than those in the control group (P>0.05, P<0.05 and P<0.05 for the low- moderate- and high-concentration intervention groups, respectively), and demonstrated APS dose-dependence. The results indicate that APS are capable of increasing adiponectin secretion and reducing IL-6 secretion by 3T3-L1 rat adipocytes in a dose-dependent manner. These findings may identify a potential mechanism for ameliorating IR using APS.

## Introduction

Insulin resistance (IR) refers to the reduced biological efficacy of insulin on insulin effector organs and the consequent decrease in glucose uptake and elimination in surrounding tissues, including the liver, skeletal muscle and adipose tissues. IR is a physiological and pathological state wherein normal or above-normal concentrations of insulin are only capable of exerting biological effects that are reduced compared with those expected in the normal range. Under IR, higher levels of insulin are required to induce a reaction comparable to that induced by normal levels of insulin, and hyperinsulinaemia or hyperproinsulinaemia, in which exogenous IR occurs, often accompany the condition. Hyperinsulinaemia is one of the predominant indicators of IR (1). Numerous pathophysiological changes occur during IR, which increase risk factors for various conditions, including diabetes, hypertension, atherosclerosis, dyslipidaemia and central obesity. Considering the high rates of morbidity and mortality associated with cardiovascular diseases, the risks associated with IR have become a widespread concern and the focus of medical research (2).

Obesity is a condition often accompanied by a chronic and subclinical inflammation that is associated with IR, insulin secretion and the development of atherosclerosis through the secretion of inflammatory cytokines. Obesity increases the incidence of type 2 diabetes mellitus (T2DM) and cardiovascular events. Inhibition of the inflammatory process associated with obesity may represent a potential pharmaceutical target for the prevention and treatment of T2DM and coronary heart disease. Numerous studies on fat cytokines initially identified obesity as a low-grade inflammatory disease. Low-grade inflammation is a key determining factor for IR, and low-grade inflammation molecules have been associated with obesity and IR (3).

Adipose tissues are important sources of inflammatory mediators (4), and adiponectin is a fat cytokine closely associated with insulin sensitivity. Numerous studies have demonstrated that, plasma adiponectin concentrations of animals and humans are negatively correlated with fasting blood glucose concentrations and insulin concentrations, that are positively correlated with insulin sensitivity. Furthermore, it has been shown that secretion of plasma adiponectin is affected by peroxisome proliferator-activated receptor γ (PPAR-γ) and insulin receptor substrate 1 (IRS-1), and that adiponectin expression is negatively correlated with interleukin (IL)-6 (5).

Astragalus polysaccharides (APS), which are capable of improving IR and reducing blood glucose levels, are key components of *Astragalus mongholicus,* which is widely used in Traditional Chinese Medicine. Studies have demonstrated that APS exert their functions through PPAR-γ and IRS-1 (6,7). The aim of this study was to investigate the effect of APS on adiponectin and IL-6 secretion by 3T3-L1 cells, and to evaluate the potential of APS for clinical diabetes treatment.

## Materials and methods

### Cell culture

3T3-L1 adipocytes were inoculated in high-glucose Dulbecco’s modified Eagle medium (DMEM; Hyclone Co., Thermo Fisher Scientific, Waltham, MA, USA) containing 100 g/l foetal calf serum at 37°C and in 5% CO_2_, in accordance with a modified Delex method. Following contact inhibition for 2 days, high-glucose DMEM (Hyclone Co.) containing 10 mg/l insulin, 200 pmol/l T3, 10 μg/ml transferrin and 0.5 mmol/l 3-isobutyl-1-methlyxanthine foetal bovine serum was used for further culture, until mature adipocytes were induced. Adipocytes were collected and assigned to intervention groups upon reaching ~90% differentiation. The present study was approved by the Ethics Committee of The Tianjin Prevention and Treatment Center of Occupational Diseases.

### Determination of cell viability

Five drops of mixed cultured cell suspension and five drops of 0.4% trypan blue solution were mixed. Cells were then counted using a haemocytometer (XB-K25, Shanghai Qiujing biochemical reagent and Instrument Co., Ltd., Shanghai, China) for 2 min, and blue-stained dead cells were observed. The rate of living cells was calculated as the ratio of living cells to the total number of cells.

### Cell growth curve

The cell suspension was seeded at a density of 1×10^5^/ml in 24-well plates and randomly divided into eight groups. The total cell number in each group was calculated, and subsequently the mean was calculated from measurements obtained from three wells. The cell counting method was based on previous cell biology experiments (8).

### Oil Red O staining

Following removal of the medium, 3T3-L1 cells were washed three times with phosphate-buffered saline (PBS), fixed in 10% formalin solution for 30 min, then washed twice with PBS and dip-stained with 5 ml Oil Red O working solution for 10 min. The resultant orange fat droplets were observed using a microscope (Olympus BX51WI-DPMC; Olympus Corporation, Tokyo, Japan). The Oil Red O working solution was subsequently removed, and the 3T3-L1 cells were washed two times and re-dyed with haematoxylin for 10 min prior further images being obtained. This method simply and rapidly reflects pre-adipocyte conversion rates cultured *in vitro*. It has been demonstrated that the accuracy and sensitivity of this method are similar to those observed for the determination of marker enzyme (glycerol phosphate dehydrogenase) during the differentiation of pre-adipocytes (9). Thus, the present method is frequently used to identify the differentiation of pre-adipocytes cultured *in vitro*.

### Evaluation of adiponectin and IL-6 levels

Following identification, 3T3-L1 cells were seeded in six-well plates at a density of 1×10^6^ and assigned to either the APS intervention groups or the control group. The final concentrations of APS (American Generalisation Pharmaceutical Company, Stanford University Science Park, Stanford, CA, USA) were 10, 0.1, 0.001 and 0 μg/μl for the high- moderate- and low-concentration groups respectively. Following incubation at 37°C and in 5% CO_2_ for 48 h, the supernatant was collected for drug testing. Concentrations of adiponectin and IL-6 were measured using enzyme-linked immunosorbent assay in accordance with the kit instructions (Shanghai Chuanfu Biotech Co., Ltd., Shanghai, China).

### Statistical analysis

All experimental data are expressed as the mean ± standard deviation. Statistical analyses were performed using SPSS 10.0 statistical software (SPSS, Inc., Chicago, IL, USA). Heterogeneous variance data were corrected by t-test, and homogeneous variance data were analyzed using one-way analysis of variance, and the abnormal distribution data using the Spearman correlation coefficient formula. A value of P<0.05 was considered to indicate a statistically significant difference.

## Results

### Morphological observation

**Figure 1:**
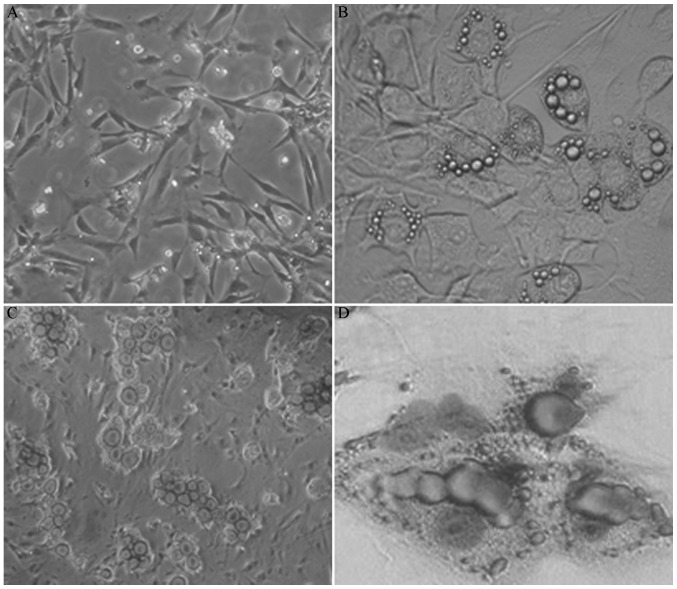
Culture and identification of 3T3-L1 cells. (A) Fiber-like growth observed three days after inoculation. (B) Formation of varing sizes of lipid droplets, 10 days after induction. (C) Oil red O staining, with which lipid droplets were stained red. (D) Hematoxylin re-staining, with which lipid droplets were stained red and nuclei were dyed blue. (A and B) magnification, ×100; (C and D) magnification ×200.

The majority of the inoculated 3T3-L1 cells exhibited characteristic shuttle shapes with fibroblast-like growth, round nuclei and sustained proliferation (Fig. 1A). Four days after induction, lipid droplets appeared within the 3T3-L1 cells. Droplets were initially focused at the cell periphery; however, the droplets gradually increased in size and spread throughout the cell. The cells subsequently became more round in morphology. Cells of various sizes containing fat droplets were formed after ~10 days (Fig. 1B) or integrated into large lipid droplets. The number of cells containing lipid droplets gradually increased with time. Following Oil Red O and haematoxylin staining, lipid droplets were stained red and nuclei were stained blue (Fig. 1C and D).

### Cell viability

The rate of living cells was ≥90%, indicating the high viability of cells obtained from enzyme digestion. These cells were thus used for subsequent culturing and monitoring.

### Cell growth curve

**Figure 2:**
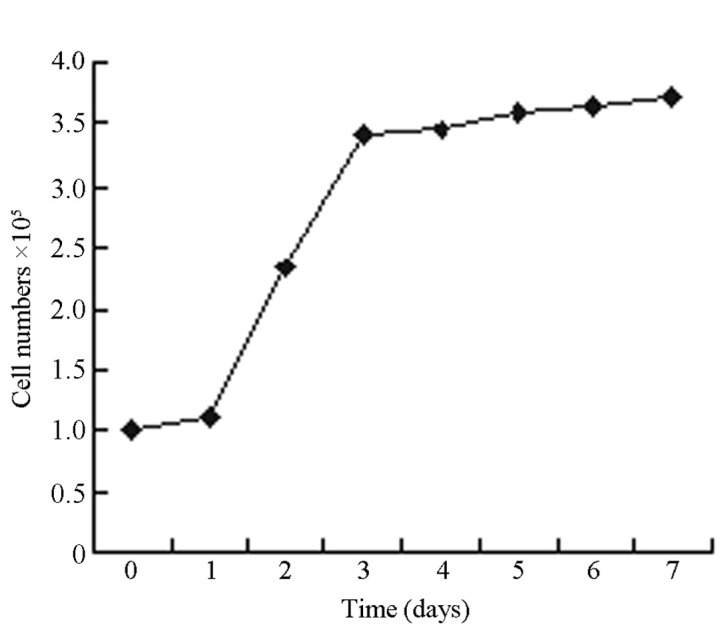
3T3-L1 cell growth curve.

Fig. 2 shows that the growth of 3T3-L1 cells formed an S-shaped curve. Proliferation exhibited a pattern of latency, followed by a period of logarithmic growth, then a plateau. The 3T3-L1 cells began to proliferate 24 h after seeding, and the number of cells increased to ~3.5 fold after 72 h. Consequently, the initial 72 h was considered to be the logarithmic growth period, during which the most rapid proliferation occurred. The 3T3-L1 cells entered the plateau phase on the fourth day after seeding. Using the Patterson formula, T_d_=ΔT × lg2/(lgN_t_/lgN_0_), (where T_d_, doubling time; ΔT, time interval; N_t_, the end point cell number; N_0_, the initial cell number) the population doubling time of 3T3-L1 cells in the logarithmic growth period was determined to be 41 h. Therefore, the second to fourth day of 3T3-L1 cell proliferation was considered to be the critical period of induction.

### Adiponectin and IL-6 secretion following APS intervention

Levels of adiponectin and IL-6 secretion by 3T3-L1 cells following stimulation with APS are shown in Table I. In comparison with the control group, the 3T3-L1 cells in the moderate-concentration group (APS, 0.1 μg/μl) demonstrated significantly increased adiponectin secretion (P=0.001). 3T3-L1 cells in the low-concentration group (APS, 0.001 μg/μl) showed significantly reduced adiponectin secretion (P<0.05), whereas the 3T3-L1 cells in the high-concentration group (APS, 10 μg/μl) demonstrated no significant reduction in adiponectin secretion (P>0.05) compared with the control group.

**Figure 3:**
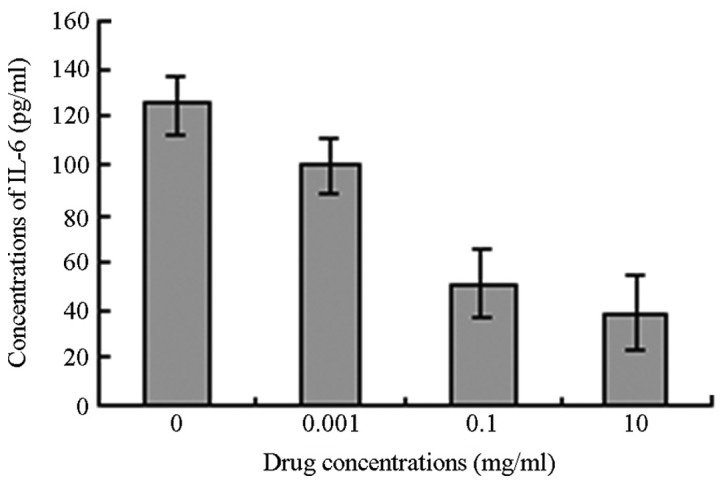
Influence of Astragalus polysaccharides on IL-6 secretion. IL-6, interleukin-6.

**Figure 4:**
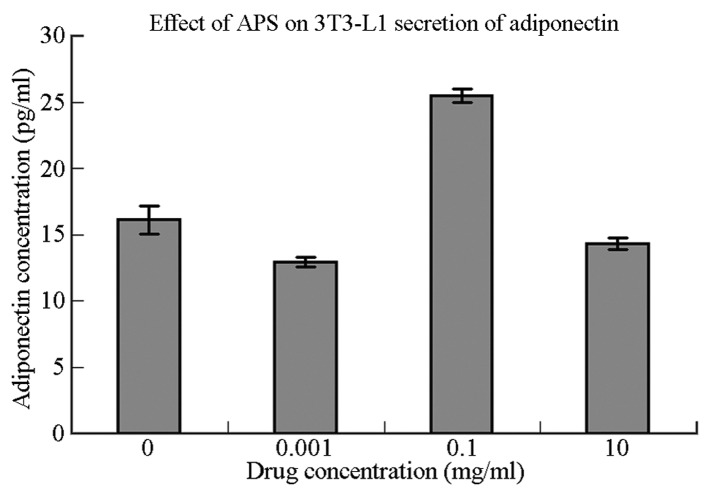
Influence of Astragalus polysaccharides on adiponectin secretion from 3T3-L1 cells.

As the concentration of APS increased, the level of IL-6 secretion by the 3T3-L1 cells was observed to decrease. While the decrease in IL-6 secretion in the low-concentration group was not considered statistically significant (P>0.05), the decreases in the moderate- and high-concentration groups were considered statistically significant compared with the IL-6 secretion by the 3T3-L1 cells in the control group (P<0.05) (Figs. 3 and 4).

## Discussion

IR refers to the phenomenon wherein the body exhibits a reduced biological response to insulin. Reductions in the biological effect of each unit of insulin in insulin effector organs result in a decrease in glucose uptake and elimination in surrounding tissues, including the liver, skeletal muscle and adipose tissues. As a pathological state, IR has a significant association with metabolic syndrome and is closely associated with obesity (10).

Found in Mongolia, *A. mongholicus* was first recorded in ‘Shen Nong’s Herbal Classic’ as a top-grade herbal plant that is sexual Gan, tepid. *A. mongholicus* is beneficial for the spleen and lungs and has a number of functions, inluding lifting yang, tonifying qi and strengthening exterior (11). APS are water-soluble macromolecular compounds with potent biological activities, and may be extracted from *A. mongholicus*. APS exert various effects, including oxygen free radical scavenging, lipid peroxidation reduction and immune regulation, and exhibit antitumour and anti-ageing properties (12).

In recent years, a number of studies have focused on the endocrine function of adipose tissue for energy-storage organs. Adipose tissue is capable of secreting numerous bioactive substances involved in various metabolic processes, including tumour necrosis factor α, plasminogen activator inhibitor-1, leptin and IL-6. 3T3-L1 cells are frequently used as a model for studying the endocrine function of adipocytes. Adiponectin, a lipid-derived plasma protein specifically secreted by fat cells, has been indicated to improve IR and exert anti-atherosclerotic and anti-inflammatory effects (13). However, the molecular mechanisms underlying the role of adiponectin in the treatment of diabetes (14) remain unclear.

*In vivo* and *in vitro* experiments have shown that the expression of adiponectin and PPAR-γ is closely associated with fat-specific nuclear receptors. Decreases in PPAR-γ transcriptional activity increase inflammatory cytokine secretion and decrease levels of adiponectin, resulting in a decrease in insulin sensitivity (15). mRNA expression and adiponectin secretion in humans, obese (ob/ob) mice and cultured 3T3-L1 cells increase following treatment with PPAR-γ agonists (16–19). PPAR belongs to the hormone nuclear receptor superfamily and is composed of three subtypes encoded by three different genes: PPAR-α, -δ and -γ. PPAR-γ is one of the most adipose tissue-specific nuclear transcription factors with important functions in the proliferation and differentiation of fat cells. PPAR-γ has also been found to increase the number of insulin receptors in fat cell membranes, thereby upregulating adiponectin expression (17).

Thiazolidinedione (TZD) is a synthetic ligand of PPAR-γ, and is traditionally used for the treatment of IR. TZD promotes glucose transport, increases adipose tissue insulin sensitivity and is used to alleviate bone loss, fluid retention and certain liver toxicities. Diabetes treatment guidelines worldwide recommend that patients decrease their sugar intake, and avoid weight gain and the problems associated with low blood sugar. However, the potential of TZD may be limited, due to its association with obesity and IR.

Given recent developments in Traditional Chinese Medicine and molecular biology, numerous Chinese medicinal ingredients have been identified to demonstrate functional similarity to PPAR agonists without significant side-effects in humans. Previous studies have demonstrated that APS increase PPAR-γ mRNA expression and promote cell differentiation, similar to TZDs. Furthermore, adiponectin secretion has been found to increase following PPAR-γ activation. In the present study, following stimulation with 0.1 μg/μl APS, 3T3-L1 adipocytes demonstrated significant increases in adiponectin secretion compared with the control group (P<0.01). These results are consistent with the effective concentrations of APS necessary for stimulation of PPAR-γ mRNA expression in fat cells reported in a previous study (2). Following stimulation with high and low concentrations of APS, adiponectin secretion decreased compared with that in the control group (P>0.05 and P<0.05, respectively), indicating that optimal APS concentrations are required to obtain the desired effect, or regulatory mechanisms that lead to a reduction in adiponectin secretion occur. A previous study suggested that APS are capable of enhancing the activity of the insulin receptor and downstream molecules of phosphatidylinositol 3-kinase (PI3K)-protein kinase B (PKB) (20), and that the sustained activation of P13K-PKB may reduce the expression of adiponectin proteins in fat cells (21).

In addition to APS, other inflammatory factors, including IL-6, have been shown to affect adiponectin secretion. IL-6 is an inflammatory cytokine with endocrine characteristics closely associated with adiponectin. Approximately one-third of IL-6 in the body is secreted by fat cells. Plasma IL-6 levels increase during IR associated with obesity and diabetes; therefore, IL-6 plasma concentration may represent an independent predictor of risk for T2DM (22). Endogenous IL-6 may interact with adiponectin through paracrine and autocrine transport mechanisms, and it has been demonstrated that IL-6 produced by local adipose tissue directly inhibits local adiponectin production (23,24).

In this study, rat 3T3-L1 adipocytes exhibited a significant concentration-dependent decrease in IL-6 secretion when treated with APS compared with the control group (P<0.01). However, levels of IL-6 secretion were not significantly correlated with adiponectin secretion (R=−0.13). This was inconsistent with the findings of Conroy (25), who identified a negative correlation between IL-6 and adiponectin. Two reasons may explain such contrasting findings: Firstly, differences between *in vivo* and *in vitro* environments may influence such contrasting results. For example, in an *in vitro* environment, differences in co-stimulatory factors, cell sources and different external stimuli are eliminated. Secondly, adiponectin secretion may be affected by multiple factors, among which IL-6 is only one. The reduced IL-6 concentrations induced by APS may represent a mechanism by which IR may be improved. Rotter *et al* (26) found that IL-6 is capable of regulating insulin transduction signals and the development of IR, by inhibiting IRS-1, glucose transporter type 4 and PI3K in cultured 3T3-L1 adipocytes (27,28). APS are considered to intervene in such pathways indicating that APS may improve IR through multiple mechanisms.

In conclusion, *in vitro* experiments demonstrated that optimal concentrations of APS are capable of reducing secretion of the inflammatory cytokine IL-6 and increasing secretion of the protective factor adiponectin, suggesting a potential mechanism by which APS may ameliorate IR in the human body. These findings may indicate a novel therapeutic approach for the clinical treatment of diabetes, using APS. However, no significant correlation was observed between APS and adiponectin levels. Additional factors that may affect adiponectin secretion, including the function and status of IL-6 and regulation by other protein factors, require further study. Previous studies have shown that APS affect the adenosine monophosphate-activated protein kinase signalling (29) and upstream inflammatory factor (30) systems. Further investigations into the physiological mechanisms of APS are required for the development of novel drugs for diabetes treatment.

## Figures and Tables

**Table I:** Effects of various concentrations of APS on IL-6 and adiponectin secretion by 3T3-L1 cells.

Concentration of APS (μg/μl)	n	Concentration of IL-6 (pg/ml)	n	Concentration of adiponectin (ng/ml)
0 (Control group)	6	125.55±12.34	6	16.12±1.06
0.001	6	100.56±11.60	6	12.92±0.37^a^
0.1	6	50.734±4.42^a^	6	25.44±0.51^a^
10	6	38.488±4.86^a^	6	14.31±0.42

a P<0.05 compared with the control group.

APS, Astragalus polysaccharides; IL-6, interleukin-6.

## Abbreviations

IR: insulin resistance
APS: Astragalus polysaccharides
IL-6: interleukin-6
IRS-1: insulin receptor substrate 1
PI3K: phosphatidylinositol 3-kinase
PKB: protein kinase B
NF-κB: nuclear factor-κ-light-chain-enhancer of activated B cells
PPAR: peroxisome proliferator-activated receptor
